# Parasites Induced Skin Allergy: A Strategic Manipulation of the Host Immunity

**DOI:** 10.4021/jocmr456w

**Published:** 2010-12-11

**Authors:** Alketa Hysni Bakiri, Ervin Cerciz Mingomataj

**Affiliations:** a University of Tirana, Nursing Faculty, Dept. of Preclinical Disciplines, Albania; b "Mother Theresa" School of Medicine, Department of Allergology and Clinical Immunology, Tirana, Albania

## Abstract

The absence of a consistent link between parasitoses and skin allergic symptoms in the clinical investigations contrasts to the fact that some parasites are the most potent inducers of immunoglobulin E that exist in nature. To shed some light into this question, this review is focused on the actual knowledge regarding parasites life cycle, interactions with host immunity, the influence on host behavior, and finally the role of all these factors on the skin allergy. The collected data demonstrate that parasites could manipulate the host behavior for its own benefit in different ways, altering its (epi)genetic, biochemical, immunologic or physiologic functions as well as altering its behavior and activity. In this context, skin allergy may be associated with certain stages of the parasites' life cycle and migration into biological barriers, but not necessarily with presence of the parasitosis in the host organism. As compared to T helper (Th) 1 response, the Th2 one, the eosinophilic infiltration and the complement inhibition could assure better conditions for the development of some parasites. Taken together, the suggested hypotheses could be a plausible explanation for the epidemiological puzzle regarding urticaria occurrence, Th2 response and parasitoses, but further studies are necessary to provide better-based conclusions.

**Keywords:**

Eosinophilic Infiltration; Host behavior; Parasites life cycle; Skin allergy; Th1/Th2 response

## Introduction

Parasitic diseases are often considered as a classic cause of urticaria [1-3]. Potential urticaria-associated pathologies can be ascaridiosis, trichinellosis, fasciolosis, giardiosis, toxocarosis, anisakiasis, schistosomosis, strongyloidosis, hydatidosis, blastocytosis, filariasis, etc [2-6].

Nevertheless, laboratory and clinical investigations greatly vary from one centre to the other and the link between these infections and skin signs does not rely on hard data. Thus, French studies have suggested a high prevalence of *Toxocara canis* markers in chronic urticaria, but anti-parasitic treatment had only inconstant effects [7]. Similarly, there are only a few case reports about cutaneous manifestations caused by giardiasis [8-10]. Many authors consider that such cutaneous manifestations as urticaria and itching were secondary to the associated gastrointestinal infection due to *Giardia lamblia* cysts and trophozoite forms, as they may disappear under specific treatment [10]. Also, the presence of urticaria associated with *Blastocystic hominis* infection has been described in very few studies [11].

The absence of a consistent link between parasitoses and skin allergic symptoms in the clinical investigations contrasts to the fact that some parasites are possibly the most potent inducers of immunoglobulin (Ig) E that exist in nature [12-16]. In a previous review, we argued about the relationship between Helminth-induced IgE response and the decrease of respiratory symptoms during this pathology [16]. In effect, the immuno-inflammatory response to helminthic infections and allergic diseases have some similarities, the most profound being the increases in eosinophils and serum total IgE concentration [12-15, 17, 18]. Both entities, helminthic infections and atopic response are Th2/interleukin (IL)-4 inducers, but helminthic infections do not only stimulate specific IgE responses against their own antigens, but also they induce a strong non-specific polyclonal synthesis of this Ig [12-16]. The experimental injection of the Ascaris-infected patients' serum into the rats' peritoneal is associated with an increase in mesentery mast cells and vascular congestion [16, 19].

In this paper we will focus in the aspect of relationship between the skin allergy and parasitic pathologies. Irrespective of the abundant literature regarding the association between exposure to parasites and the enhancement of IgE response, no definite conclusions about the causality of the weak association between these findings and the low frequency of urticarial reactions are yet warranted. To shed some light into this question, this review is focused on the actual knowledge about parasites life cycle, interactions with host immunity, the influence on host behavior as well as the role of these factors on the urticaria and skin allergy.

## The Role of Host-parasite Interaction in the Relationship Parasitosis-Urticarial Reaction

### Th2 host response and parasites survival/development

Parasites are designed by evolution to invade the host and survive in its organism until they are ready to reproduce [20]. They can release a variety of molecules that help them to penetrate the defensive barriers and avoid the immune attack of the host. In this respect, particularly interesting are enzymes and their inhibitors secreted by the parasites [13, 14, 20]. Thus, while serine-, aspartic-, cysteine-, and metalloproteinases are involved in tissue invasion and extracellular protein digestion, helminths secrete serpins, aspins, and cystatins to inhibit proteinases, both of the host and their own. Proteinases and their inhibitors, as well as helminth homologues of cytokines and molecules containing phosphorylcholine, influence the immune response of the host biasing it towards the "anti-inflammatory" Th2 type [13-15, 20]. Besides the eosinophilic infiltration, the IgE response as component of the Th2 profile is estimated to be a cornerstone of host defense during parasitoses [12-16, 21].

### During parasitoses, the efficacy of the Th1 response may be superior to the Th2 one

Current reports suggest that interaction between parasites and hostile immunity is more complex than previously estimated. In this respect, the experimentally obtained data indicate that even hostile cytokines used for cell-cell communication can also be exploited by the parasite as clues to find suitable target organs [22]. In nematodes, the Th2 type response is affected by parasite dose [21]. For Trichiuris muris infections, Th1-type immune responses occurred in animals given repeated low dose infections; latterly, the immune response developed into a protective Th2-type response. During *Strongyloides ratti* infections, the host immune response changes both qualitatively from a Th1- to a Th2-type immune response and the Th2-type response increases quantitatively with higher dose infections [21]. Furthermore, parasite survivorship was significantly negatively related to the concentration of parasite-specific IgG1 and IgA [23, 24]. At the metacestode stage of Echinococcus infection, studies of the immune responses in the experimental murine model as well as in humans have shown that (i) cellular immunity induced by a Th1-type cytokine secretion was able to successfully kill the metacestode at the initial stages of development; (ii) antigenic proteins and carbohydrates of the oncosphere/metacestode were able to interfere with antigen presentation and cell activation, leading to the production of IL-10 and other mediators by host lymphocytes and other immune cells, and therefore, to the inhibition of the effector phase of cellular immune reaction; and (iii) immunogenetic characteristics of the host were essential to this parasite-induced deviation of the immune response [25]. Regarding anisakiasis, acute symptoms are caused by an IgE-mediated allergic reaction in the gastrointestinal wall. Cuellar et al. demonstrated that anisakis antigens react with antibodies raised against vertebrate IL-4 [26]. With respect to schistosomosis, most of the chronic patients presented a Th2 profile with low production of gamma interferon (IFN-γ) as compared to subjects resistant to this infection, while the intensity of infection favors the production of IL-10 [27]. In addition, the blockade of IL-4 and IL-5 as well as the addition of the recombinant IL-10 significantly reduced the peripheral blood mononuclear cell proliferative response to soluble egg and adult worm antigens [28]. Meanwhile, experiments in mice have shown that the relative success of *Giardia muris* in completing its life cycle in a primary infection might be due, in part, to the stimulation of a Th2-type response. In contrast, a stronger Th1 response may lead to a better control of the primary infection [29]. These data suggest that IL-10 is an important cytokine in regulating the immune response and possibly controlling morbidity in human parasitoses, and that the production of IFN-γ may be associated with resistance to infection [28].

Taken together, these findings may suggest that hostile IgE/Th2 response has defensive effects, but the IgG/Th1 type may also provide such qualities, which in some situations seem to be superior to the Th2 one. In vivo, the Th2 profile might be not simply a host-chosen reaction, but rather the most efficient permitted humoral response during host-parasite interaction. The fatal outcome in apparently immunocompetent patients due to multiorgan failure after *Strongyloides stercoralis* septicaemia following a short course of prednisolone therapy may lead to the suggestion that glucocorticoids may suppress the parasite-attenuated host immune defenses [30]. In our opinion, the Th2 deviation may permit parasites to invade the host organism, and to select specific organs or host cell types as predilection site to reside, maturate or even proliferate [13, 31, 32]. While many microparasites escape immune attack by antigenic variation or sequestration in specialized niches, helminths appear to thrive in exposed extracellular locations, such as the lymphatics, bloodstream, or gastrointestinal tract. Key events among the host cell population are dominance of the Th2 cell phenotype and the selective loss of effector activity, against a background of regulatory T cells, alternatively activated macrophages, and Th2-inducing dendritic cells. The sum effect of these changes to host reactivity is to create an "anti-inflammatory" environment, which is most favorable to parasite survival [33]. In *Echinococcus multilocularis* infection, a combined Th1 and Th2 cytokine profile appears crucial for prolonged metacestode growth and survival. Vuitton has demonstrated that Th1 cytokines promote the initial cell recruitment around the metacestode and are involved in the chronicity of the cell infiltrate leading to a fully organized periparasitic granuloma and its consequences, fibrosis and necrosis [25]. Meanwhile, the Th2 cytokines could be responsible for the inhibition of a successful parasite killing, especially because of the "anti-inflammatory" potency of IL-10. This combination of various arms of the immune response results in a partial protection of both Echinococcus metacestode and host [25, 34]. However, it may also be considered responsible for several complications of the disease. The Th2-related IgE synthesis and mast cell activation, well known to be responsible for anaphylactic reactions in cystic echinococcosis, are more rarely involved in 'allergic' complications in alveolar echinococcosis [25]. With regards to *Anisakis simplex*, it shares several epitopes with IL-4, important for the Th2 response development in human anisakiasis, where the parasite may modulate the Th1-Th2 dichotomy for its own benefit by mucosal inflammation control in an attempt to avoid the larval expelling [26].

### An additional factor of the IgE response induction: the inhibition of complement pathway

Apart from the increasing of the tissue permeability and larvae penetration, the induction of IgE response may have an additional advantage for the development of parasites in the hostile organism. In contrast to IgG, the IgE antibody does not activate the complement system. In animal experiments, IgG is shown to activate complement, and therefore, to kill the L3 larvae of *Angiostrongylus cantonensis* [35]. In vivo, however, the classic pathway activation can be avoided because IgE does not interact with fraction C1 of the complement [36]. Regarding the complement inhibition in humans, the larval L3 products of anisakis exercised a stronger effect on the classical pathway than on the alternative one, constituting a mechanism to evade host defenses, similarly to other parasitic diseases. In this context, detailed studies revealed that larval products of *Anisakis simplex* act at the level of the C3 and C2 proteins, which are early components of the classical complement pathway [37, 38]. These findings suggest that parasites cannot "switch off" the humoral host immunity, but they could induce the Th2 profile. The Th2/IgE response may assure better survival possibilities for the parasites within the host due to parasitic avoidance of the complement pathway.

### Urticaria as symptom of parasite migration through the biological barriers

Apart from parasitoses, the IgE response is also strongly associated with pathogenesis of the immediate allergic diseases such as urticaria, angioedema, etc. Despite expectations, the association of the skin allergic reactions with presence of parasitic infections does not rely on hard data [7]. Recently, much evidence is collected about the interaction's details between the hosts and parasites, but fewer attempts are made to clarify the urticarial puzzle during parasitoses. Reflecting on these findings, it could be mentioned that urticaria is a skin manifestation, related to helminths or arthropods with a cutaneous phase: Schistosoma, *Sarcoptes scabiei*, as well as ticks and other blood sucking arthropods have been involved in Th2-based immunologic mechanisms [39, 40]. Among patients with toxocaral infection, an elevated ECP level was significantly associated with both cough and rhinitis, a high level of specific anti-toxocara IgE with itchy rashes [41]. Loeffler's syndrome, which resembles the pathophysiological features of chronic asthma with its Th2-related immunologic feature, is related to ascaris and necator infection, both of which have an obligatory pulmonary phase [42]. Some helminths like necator and schistosoma have even both a cutaneous and pulmonary phase [43]. Such pathologies as larva migrans or cercarial dermatitis are also examples of the skin migration. Being attempts to find the suitable host environment, the parasitic induction of urticaria, atopic phenotype, itching and the increased tissue permeability could favorise larvae migration and therefore, the completing of the parasitic life cycle [13, 16]. In the case of human anisakiasis, this would be a hopeless attempt to destroy hostile barriers (intestinal wall, etc) to search for the missed suitable environment, because they cannot develop within terrestrial mammalians. Consequently, the type I allergic reaction takes at least 2 to 6 hours to be triggered by alive larvae, while the ingestion of lyophilized larvae, or its equivalent in antigen, does not induce clinical symptoms in sensitized individuals [44, 45]. A similar scenario develops also within paratenic hosts during larvae migration in different visceral organs, like in case of *Toxocara canis* [46]. These data suggest that the development of allergic symptoms could be an active effect of parasites and not only a host defense reaction.

In some particular cases, IgE and IgG values will differ depending on the time relapsed between the parasitic contact and therefore on its developing phase [47]. During infection of mice with *Litomosoides sigmodontis*, female adult worms from prepatent infections protects mice injected with lipopolysaccharide due to inhibition of the host Th1 response, whereas microfilariae worsen lipopolysaccharide-induced sepsis through the induction of the Th1-related cytokines in the peripheral blood [31]. Similarly to the immune modulation, Giardia lamblia can express different kinds of variant surface proteins (VSP). The giardial variant-type formation and VSP mRNA levels after infection of mice with cysts lead to an antigenic reset of the parasite, which appears to be associated with excystation [48]. In this respect, the VSP H7 type has to be regarded as a predominant variant of *Giardia lamblia* clone GS/M-83-H7 that (re-)emerges during early-stage infection and may contribute to an optimal establishment of the parasite within the intestine of the experimental murine host [48]. In summary, the Th2 response seems to be a host reaction, induced under the parasites' influence. It may permit the migration of parasites under the skin, in lymphatic ways and into some parenchimatous organs. In a few cases, this response may be induced in some developing phases, such as in case of excystation (which is also a kind of barrier penetration) or epithelial inoculation of giardiasis. Taken together, these data indicate that urticarial symptoms may be related to the larval stage or hostile tissue penetration, but not necessarily only to the presence of parasitic infection in the human organism. This may explain the lack of clear evidence regarding the correlation between the parasitic diseases and the urticaria development.

### Eosinophils as barrier perforators: are they double game players?

In spite of the humoral mechanisms, there is evidence of important parasite-induced effects on innate cell types, particularly mast cells and eosinophils. According to Maizels et al., the sum effect of these changes to host reactivity is to create an "anti-inflammatory environment", which is most favorable to parasite survival [15, 33]. However in our opinion, the role of eosinophils is more complex. The eosinophils like the complement system can induce increased cell membrane permeability [49, 50]. This eosinophil-induced role is also shown on various biologic barriers, including the parasite surfaces. This effect is called "frustrated phagocytosis" [48, 51]. Thus, Kaji et al. reported about a case of urticaria, eosinophilic cholecystitis and a simultaneous onset with pericarditis after an ascaris infection [52]. Infection from *Angiostrongylus cantonensis* is generally associated with damage of blood-brain barrier and neurological disorders, which is assumed to be a consequence of eosinophilic meningitis [50, 53].

Besides the host-influence, eosinophils migration close to parasites could be also a strategic step induced even from the parasite, leading to the allergic symptoms. While a hypereosinophilia is an argument in favor of a progressive toxocara infection, high total IgE level is considered a hallmark of visceral infections by parasites [54, 55]. Furthermore, anisakis larvae extract exercises a chemotactic effect for eosinophils [56]. In this context, alive L3 larvae can exhibit the main hyperergic response in the duodenum, decelerating their transit into the successive parts of intestinum, but also inducing the transit into the tissues outside the duodenal lumen [57]. In other words, since parasites affect the behavior traits with selectively benefit the parasite, rather than causing a general alteration of the host behavior, the induction of the urticaria and the atopic phenotype might be only an efficient or hopeless larval attempt to find the suitable host to produce eggs. The IgE-response, the eosinophilic chemotaxis, or the general itching cannot be only host defenses, but also larval attempts to destroy hostile barriers to search for the missed suitable hostile environments. Taken together, these findings indicate that eosinophils as biological barrier perforators are implicated under the simultaneous influence of the host and parasites in a double game. This hypothesis is supported for example by the presence of local eosinophil infiltration in the skin when *Dracunculus medinensis* larvae emerge from the inferior limbs in the ponds water [58]. In these circumstances, the eosinophils could help parasites to destroy the skin integrity, because in this stage dracunculae larvae can be developed only within thermocyclops living in ponds. This also demonstrates that helminths display highly complex life cycles in which the establishment of adults or larvae within host target organs as well as the transition of one developmental stage to the following is influenced by host-derived factors [22].

### Parasites as efficient manipulators of the host behavior

The parasite-manipulated involvement of host immune mechanisms supports the opinion that *parasites are efficient manipulators of the host behavior* (a further dimension of the parasite influence on the host reaction) [57]. The parasitic ability to affect the behavior of infected host has been documented and reviewed by different authors [13-16, 59, 60]. Although changes in the behavior of infected hosts do occur for pathogens with direct life cycle, they are most commonly recorded in the intermediate hosts of parasites with complex life cycle. In the simplest case, the changes in behavior increase rates of contact between infected and susceptible conspecific hosts, whereas in the more complex cases fairly sophisticated manipulations of the host's behavioral repertory are achieved [59-62]. In this context, because sexual reproduction of *Toxoplasma gondii* can be accomplished only in felines, there are strong selective pressures on the parasite to evolve mechanisms to enhance transmission from the intermediate host to the definitive feline host and thereby complete its life cycle. The predilection of *Toxoplasma gondii* for the brain of its intermediate host places it in a privileged position to cause such manipulation [62]. Ferreira et al. recently demonstrated that the host cell transcriptome, including the expression of distinct host cell genes, can trigger bradyzoite development and cyst formation, strongly indicating that the complex cellular environment may govern the developmental differentiation of this protozoa [63]. Moreover, the pattern of histone H3 arginine methylation distinguishes certain promoters, illustrating the complexity of the histone modification machinery in toxoplasmosis [64, 65]. Being placed in the intermediate host brain, the toxoplasma-expressed epigenomic mechanisms may lead to variations in gene expression during the transformation of tachisoites into bradysoite, waiting then for the definitive host. This way, *Toxoplasma gondii* dispose the ability to manipulate the personality profile of the intermediate host [16, 59]. The toxoplasmosis-infected people are more predisposed to take a risk, or are less watchful for example in the motorways, whereas toxoplasmosis-infected rats can even lose the cat predation risk [16, 61, 66, 67]. Also the loss of predation risk by rats or the loss of watchfulness by humans at least at the prehistoric time before the invention of entombment, after a toxoplasmic infection, led usually to the rip of their bodies from some carnivore and therefore to the transmission of the parasite into its definitive host like felines [16]. The parasite thus manipulates the behavior of its intermediate host to enhance its transmission to the definitive one [66, 67]. In a similar manner, the experimentally *Toxocara canis*-infected BALB/c mice take significantly longer to drink from a water source compared with control mice [68]. Moreover, infected mice displayed reduced levels of anxiety to aversive and exposed areas of the maze, particularly in the case of the moderate and high intensity mice [69]. These findings suggest that a toxocara-infected paratenic host can be an easier prey for their predators. During dracunculiasis, the burning effect in patient's lower limbs during pregnant larvae extrusion is also a host behavior manipulation, because the expelling first-stage larvae can be developed only within copepods of the ponds [58]. Consequently, the patient hurries to immerse the burning limbs in the ponds in order to cool them.

The reduction of respiratory allergic symptoms (like wheezing or airway hyperreactivity) in intensive helminth-infected populations is another example of host behavior manipulation and an evolutionary adaptation from the point of view of parasites [16]. This reduction assures those better chances for their reproduction and development in the environment "host", because the liberation mammalian efforts against these parasites are suppressed. Thus, toxocara, ascaris, trichiuris, and hookworm have a phase of larval migration into the respiratory system or at least, their entrance way (as eggs) in the human body is the nose or the mouth [16, 42]. To assure their penetration into the host and latter their reproduction or development, these parasites need to affront or avoid the reactive (including allergic) response of the host (like the cough, airway obstruction and airway hyper-responsiveness) due to induction of immuno-modulatory network [13-16, 70].

The manipulation of host reaction is not an exclusive ability of parasites. Common respiratory infective pathogens can manipulate the host behavior. While during incubation they could suppress pathologic symptoms due to inhibition of the innate immunity, later they might anticipate the providing of specific humoral immunity abandoning the host due to induction of the respiratory symptoms [71]. Thus, the soluble G glycoprotein acts as bacterial cytokine with inhibiting expression of ICAM-1, IL-8 and NF-κB during incubatory period of respiratory syncytial virus (RSV) [71-74]. The experimental infection of BALB/c mice with a RSV mutant lacking the glycoprotein G gene increases NK and neutrophil trafficking to the lungs compared to control mice infected with a strain of RSV that has glycoprotein G [71, 73]. Although the secreted form of glycoprotein G accounts for no more than 20% of the total glycoprotein synthesized in cell culture through the course of infection, secreted glycoprotein represents about 80% of the protein released into the medium early in infection, during the first 24 h [73, 74]. This scenario first could assure a maximal multiplication for the infectious agents; then the host abandonment on time to catch a next one assures maximal successive reproduction [71]. Taken together, these data demonstrate that parasites and other infective agents can be efficient host manipulators using them for their reproductive success, independently to the fact if they induce or inhibit the host pathology [16, 50, 71].

**Figure 1.**
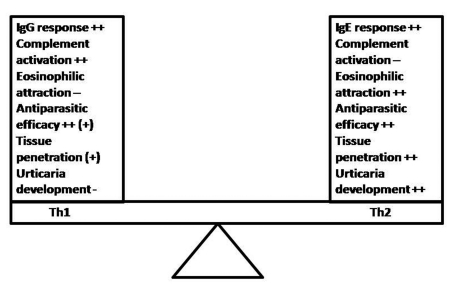
Th1 vs. Th2 response during parasitoses and the development of allergic skin symptoms: Although both responses provide antiparasitic effects, the Th1 response seems to be superior to the Th2 one. Maybe the Th2 response is a host-response, chosen by the parasite that is associated with better survival and hostile tissue penetration. The eosinophil chemotaxis and the avoidance of complement-dependent innate mechanisms are targets of parasite-induced host immune modulation in order to improve its development and survival possibilities within hostile organism.

## Conclusions

Based on the current knowledge, it could be concluded that parasites try to manipulate the host behavior for its own benefit in different ways, altering its (epi)genetic, biochemical, immunologic or physiologic functions as well as altering its behavior and activity [13-16, 62, 65, 75, 76]. Current data indicate that skin allergy may be associated with certain stages of the parasites' life cycle, but not necessarily with presence of the parasitosis in the host organism. As compared to Th1 response, the Th2 one (including the IgE production), the eosinophilic infiltration and the complement inhibition might assure better conditions for the development of some parasites (Fig. 1). The ambiguity of the host immune response during parasitic infection remains a puzzle, but much evidence stresses the fact that the sum effect of the deviated host reactivity may create an environment, which is also favorable for the parasite survival [33]. Taken together, the combination of suggested hypotheses could be a plausible explanation for the epidemiological association's paradox between skin allergy (including urticaria), IgE response and parasitoses [10]. Nevertheless, further studies focused on the stages of parasites' development may lead to the providing of better-based conclusions and invention of novel therapeutic strategies [16, 70]. They can consist on the monitoring of experimental parasitic development or dispersion/penetration on the host tissue and the association of parasitic life stages with urticarial development.

## References

[1] Ronellenfitsch U, Bircher A, Hatz C, Blum J (2007). [Parasites as a cause of urticaria. Helminths and protozoa as triggers of hives?]. Hautarzt.

[2] De Gentile L, Grandiere-Perez L, Chabasse D (1999). [Urticaria and Parasites]. Allerg Immunol (Paris).

[3] Pasqui AL, Savini E, Saletti M, Guzzo C, Puccetti L, Auteri A (2004). Chronic urticaria and blastocystis hominis infection: a case report. Eur Rev Med Pharmacol Sci.

[4] Giacometti A, Cirioni O, Antonicelli L, D'Amato G, Silvestri C, Del Prete MS, Scalise G (2003). Prevalence of intestinal parasites among individuals with allergic skin diseases. J Parasitol.

[5] Padgett JJ, Jacobsen KH (2008). Loiasis: African eye worm. Trans R Soc Trop Med Hyg.

[6] Lapkova E, Rubik I, Vanista J, Tesarova J (1990). [Larval stages of the filaria Mansonella (Dipetalonema) perstans in a student from Africa]. Cas Lek Cesk.

[7] Cribier B, Noacco G (2003). [Chronic urticaria and infectious diseases]. Ann Dermatol Venerol.

[8] Oberholzer C, Nüesch R, Häusermann P (2007). [Urticaria and parasites: case report and general view over the most common pathogens of chronic urticaria]. Praxis (Bern 1994).

[9] Clyne CA, Eliopoulos GM (1989). Fever and urticaria in acute giardiasis. Arch Intern Med.

[10] Nenoff P, Domula E, Willing U, Herrmann J (2006). [Giardia lamblia--cause of urticaria and pruritus or accidental association?]. Hautarzt.

[11] Micheloud D, Jensen J, Fernandez-Cruz E, Carbone J (2007). [Chronic angioedema and blastocystis hominis infection.]. Rev Gastroenterol Peru.

[12] Lynch NR, Hagel IA, Palenque ME, Di Prisco MC, Escudero JE, Corao LA, Sandia JA (1998). Relationship between helminthic infection and IgE response in atopic and nonatopic children in a tropical environment. J Allergy Clin Immunol.

[13] Moreau E, Chauvin A (2010). Immunity against Helminths: Interactions with the host and the intercurrent infections. J Biomed Biotechnol 2010.

[14] Figueiredo CA, Barreto ML, Rodrigues LC, Cooper PJ, Silva NB, Amorim LD, Alcantara-Neves NM (2010). Chronic intestinal helminth infections are associated with immune hyporesponsiveness and induction of a regulatory network. Infect Immun.

[15] Maizels RM (2005). Infections and allergy - helminths, hygiene and host immune regulation. Curr Opin Immunol.

[16] Mingomataj EC, Xhixha F, Gjata E (2006). Helminths can protect themselves against rejection inhibiting hostile respiratory allergy symptoms. Allergy.

[17] Buijs J, Egbers MW, Lokhorst WH, Savelkoul HF, Nijkamp FP (1995). Toxocara-induced eosinophilic inflammation. Airway function and effect of anti-IL-5. Am J Respir Crit Care Med.

[18] Wang CC, Nolan TJ, Schad GA, Abraham D (2001). Infection of mice with the helminth Strongyloides stercoralis suppresses pulmonary allergic responses to ovalbumin. Clin Exp Allergy.

[19] Martin ES (1976). [Increase of mast cells in the peritoneal cavity of rats treated with atopic patient's serum]. Allergol Immunopathol (Madr).

[20] Dzik JM (2006). Molecules released by helminth parasites involved in host colonization. Acta Biochim Pol.

[21] de Macedo Soares MF, de Macedo MS (2007). Modulation of anaphylaxis by helminth-derived products in animal models. Curr Allergy Asthma Rep.

[22] Brehm K, Spiliotis M (2008). The influence of host hormones and cytokines on Echinococcus multilocularis signalling and development. Parasite.

[23] Paterson S, Wilkes C, Bleay C, Viney ME (2008). Immunological responses elicited by different infection regimes with Strongyloides ratti. PLoS One.

[24] Bleay C, Wilkes CP, Paterson S, Viney ME (2007). Density-dependent immune responses against the gastrointestinal nematode Strongyloides ratti. Int J Parasitol.

[25] Vuitton DA (2003). The ambiguous role of immunity in echinococcosis: protection of the host or of the parasite?. Acta Trop.

[26] Cuellar C, Perteguer MJ, Rodero M (2001). Presence of IL-4-like molecules in larval excretory-secretory products and crude extracts from Anisakis simplex. Scand J Immunol.

[27] Caldas IR, Campi-Azevedo AC, Oliveira LF, Silveira AM, Oliveira RC, Gazzinelli G (2008). Human schistosomiasis mansoni: immune responses during acute and chronic phases of the infection. Acta Trop.

[28] Correa-Oliveira R, Malaquias LC, Falcao PL, Viana IR, Bahia-Oliveira LM, Silveira AM, Fraga LA (1998). Cytokines as determinants of resistance and pathology in human Schistosoma mansoni infection. Braz J Med Biol Res.

[29] Djamiatun K, Faubert GM (1998). Exogenous cytokines released by spleen and Peyer's patch cells removed from mice infected with Giardia muris. Parasite Immunol.

[30] Ghosh K (2007). Strongyloides stercoralis septicaemia following steroid therapy for eosinophilia: report of three cases. Trans R Soc Trop Med Hyg.

[31] Hubner MP, Pasche B, Kalaydjiev S, Soboslay PT, Lengeling A, Schulz-Key H, Mitre E (2008). Microfilariae of the filarial nematode Litomosoides sigmodontis exacerbate the course of lipopolysaccharide-induced sepsis in mice. Infect Immun.

[32] Gottstein B, Piarroux R (2008). Current trends in tissue-affecting helminths. Parasite.

[33] Maizels RM, Balic A, Gomez-Escobar N, Nair M, Taylor MD, Allen JE (2004). Helminth parasites--masters of regulation. Immunol Rev.

[34] Vuitton DA (2004). Echinococcosis and allergy. Clin Rev Allergy Immunol.

[35] Shaio MF, Hou SC, Chen JG, Wu CC, Yang KD, Chang FY (1990). Immunoglobulin G-dependent classical complement pathway activation in neutrophil-mediated cytotoxicity to infective larvae of Angiostrongylus cantonensis. Ann Trop Med Parasitol.

[36] Gadjeva MG, Rouseva MM, Zlatarova AS, Reid KB, Kishore U, Kojouharova MS (2008). Interaction of human C1q with IgG and IgM: revisited. Biochemistry.

[37] Garcia-Hernandez P, Rodero M, Cuellar C (2007). Anisakis simplex: the activity of larval products on the complement system. Exp Parasitol.

[38] Garcia-Hernandez P, Rodero M, Cuellar C (2009). Study of the effect of Anisakis simplex larval products on the early and late components in the classical complement pathway. J Parasitol.

[39] Torres VM (1976). Dermatologic manifestations of Schistosomiasis mansoni. Arch Dermatol.

[40] Bruschi F (1992). The significance of the "lung phase" in host-helminth relations. Parassitologia.

[41] Magnaval JF, Faufingue JH, Morassin B, Fabre R (2006). Eosinophil cationic protein, specific IgE and IgG4 in human toxocariasis. J Helminthol.

[42] Cooper PJ (2004). Intestinal worms and human allergy. Parasite Immunol.

[43] Mulcahy G, O'Neill S, Fanning J, McCarthy E, Sekiya M (2005). Tissue migration by parasitic helminths - an immunoevasive strategy?. Trends Parasitol.

[44] Sastre J, Lluch-Bernal M, Quirce S, Arrieta I, Lahoz C, Del Amo A, Fernandez-Caldas E (2000). A double-blind, placebo-controlled oral challenge study with lyophilized larvae and antigen of the fish parasite, Anisakis simplex. Allergy.

[45] Daschner A, Alonso-Gomez A, Cabanas R, Suarez-de-Parga JM, Lopez-Serrano MC (2000). Gastroallergic anisakiasis: borderline between food allergy and parasitic disease-clinical and allergologic evaluation of 20 patients with confirmed acute parasitism by Anisakis simplex. J Allergy Clin Immunol.

[46] Sasmal NK, Acharya S, Laha R (2008). Larval migration of Toxocara canis in piglets and transfer of larvae from infected porcine tissue to mice. J Helminthol.

[47] Weiss ST (2000). Parasites and asthma/allergy: what is the relationship?. J Allergy Clin Immunol.

[48] von Allmen N, Bienz M, Hemphill A, Muller N (2004). Experimental infections of neonatal mice with cysts of Giardia lamblia clone GS/M-83-H7 are associated with an antigenic reset of the parasite. Infect Immun.

[49] Lacy P, Weller PF, Moqbel R (2001). A report from the International Eosinophil Society: eosinophils in a tug of war. J Allergy Clin Immunol.

[50] Mingomataj EC (2008). Eosinophil-induced prognosis improvement of solid tumors could be enabled by their vesicle-mediated barrier permeability induction. Med Hypotheses.

[51] Egesten A, Calafat J, Janssen H, Knol EF, Malm J, Persson T (2001). Granules of human eosinophilic leucocytes and their mobilization. Clin Exp Allergy.

[52] Kaji K, Yoshiji H, Yoshikawa M, Yamazaki M, Ikenaka Y, Noguchi R, Sawai M (2007). Eosinophilic cholecystitis along with pericarditis caused by Ascaris lumbricoides: a case report. World J Gastroenterol.

[53] Lee JD, Tsai LY, Chen CH, Wang JJ, Hsiao JK, Yen CM (2006). Blood-brain barrier dysfunction occurring in mice infected with Angiostrongylus cantonensis. Acta Trop.

[54] Humbert P, Buchet S, Barde T (1995). [Toxocariasis. A cosmopolitan parasitic zoonosis.]. Allerg Immunol.

[55] Petithory JC (2007). [Visceral and cutaneous larva migrans.]. Rev Prat.

[56] Tanaka J, Torisu M (1978). Anisakis and eosinophil. I. Detection of a soluble factor selectively chemotactic for eosinophils in the extract from Anisakis larvae. J Immunol.

[57] Sanchez-Monsalvez I, de Armas-Serra C, Bernadina W, Rodriguez-Caabeiro F (2003). Altered autonomic control in rat intestine due to both infection with Anisakis simplex and incubation with the parasite's crude extract. Dig Dis Sci.

[58] Cairncross S, Muller R, Zagaria N (2002). Dracunculiasis (Guinea worm disease) and the eradication initiative. Clin Microbiol Rev.

[59] Havlicek J, Gasova ZG, Smith AP, Zvara K, Flegr J (2001). Decrease of psychomotor performance in subjects with latent 'asymptomatic' toxoplasmosis. Parasitology.

[60] Dobson AP (1988). The population biology of parasite-induced changes in host behavior. Q Rev Biol.

[61] Berdoy M, Webster JP, Macdonald DW (2000). Fatal attraction in rats infected with Toxoplasma gondii. Proc Biol Sci.

[62] Webster JP (2007). The effect of Toxoplasma gondii on animal behavior: playing cat and mouse. Schizophr Bull.

[63] Ferreira da Silva Mda F, Barbosa HS, Gross U, Luder CG (2008). Stress-related and spontaneous stage differentiation of Toxoplasma gondii. Mol Biosyst.

[64] Gissot M, Kim K (2008). How epigenomics contributes to the understanding of gene regulation in Toxoplasma gondii. J Eukaryot Microbiol.

[65] Gissot M, Kelly KA, Ajioka JW, Greally JM, Kim K (2007). Epigenomic modifications predict active promoters and gene structure in Toxoplasma gondii. PLoS Pathog.

[66] Smith JE (2009). Tracking transmission of the zoonosis Toxoplasma gondii. Adv Parasitol.

[67] Zimmer C (2000). EVOLUTION: Parasites Make Scaredy-Rats Foolhardy. Science.

[68] Hamilton CM, Stafford P, Pinelli E, Holland CV (2006). A murine model for cerebral toxocariasis: characterization of host susceptibility and behaviour. Parasitology.

[69] Cox DM, Holland CV (2001). Relationship between three intensity levels of Toxocara canis larvae in the brain and effects on exploration, anxiety, learning and memory in the murine host. J Helminthol.

[70] Dittrich AM, Hamelmann E (2009). [Parasites and allergies: from mice to men]. Allergo J.

[71] Mingomataj EC, Rudzeviciene O (2007). From latent incubation launched into hostile symptomatic pathology: A probable survival strategy for common respiratory infectious agents. Med Hypotheses.

[72] Arnold R, Konig B, Werchau H, Konig W (2004). Respiratory syncytial virus deficient in soluble G protein induced an increased proinflammatory response in human lung epithelial cells. Virology.

[73] Polack FP, Irusta PM, Hoffman SJ, Schiatti MP, Melendi GA, Delgado MF, Laham FR (2005). The cysteine-rich region of respiratory syncytial virus attachment protein inhibits innate immunity elicited by the virus and endotoxin. Proc Natl Acad Sci U S A.

[74] Johnson PR, Spriggs MK, Olmsted RA, Collins PL (1987). The G glycoprotein of human respiratory syncytial viruses of subgroups A and B: extensive sequence divergence between antigenically related proteins. Proc Natl Acad Sci U S A.

[75] Read AF, Skorping A (1995). The evolution of tissue migration by parasitic nematode larvae. Parasitology.

[76] Berdoy M, Webster JP, Macdonald DW (1995). Parasite-altered behaviour: is the effect of Toxoplasma gondii on Rattus norvegicus specific?. Parasitology.

